# Discrete Element Method Modeling for the Failure Analysis of Dry Mono-Size Coke Aggregates

**DOI:** 10.3390/ma14092174

**Published:** 2021-04-23

**Authors:** Alireza Sadeghi-Chahardeh, Roozbeh Mollaabbasi, Donald Picard, Seyed Mohammad Taghavi, Houshang Alamdari

**Affiliations:** 1 Aluminum Research Centre–REGAL, Mining, Material, and Metallurgy Engineering Department, Université Laval, 1065 Avenue de la Médecine, Québec, QC G1V 0A6, Canada; alireza.sadeghi-chahardeh.1@ulaval.ca (A.S.-C.); roozbeh.mollaabbasi.1@ulaval.ca (R.M.); 2 Eddyfi Technologies Company, 3425 Rue Pierre-Ardouin, Québec, QC G1P 0B3, Canada; dpicard@eddyfi.com; 3 Chemical Engineering Department, Université Laval, 1065 Avenue de la Médecine, Québec, QC G1V 0A6, Canada; seyed-mohammad.taghavi@gch.ulaval.ca

**Keywords:** carbon anode production, crack generation, discrete element method, failure analysis, second-order work criterion, strain localization

## Abstract

An in-depth study of the failure of granular materials, which is known as a mechanism to generate defects, can reveal the facts regarding the origin of the imperfections, such as cracks in the carbon anodes. The initiation and propagation of the cracks in the carbon anode, especially the horizontal cracks below the stub-holes, reduce the anode efficiency during the electrolysis process. The failure analysis of coke aggregates can be employed to determine the appropriate recipe and operating conditions in order to avoid the formation of cracks in the carbon anodes. In this paper, it will be shown that a particular failure mode can be responsible for the crack generation in the carbon anodes. The second-order work criterion is employed to analyze the failure of the coke aggregate specimens and the relationships between the second-order work, the kinetic energy, and the instability of the granular material are investigated. In addition, the coke aggregates are modeled by exploiting the discrete element method (DEM) to reveal the micro-mechanical behavior of the dry coke aggregates during the compaction process. The optimal number of particles required for the failure analysis in the DEM simulations is determined. The effects of the confining pressure and strain rate as two important compaction process parameters on the failure are studied. The results reveal that increasing the confining pressure enhances the probability of the diffusing mode of the failure in the specimen. On the other hand, the increase of strain rate augments the chance of the strain localization mode of the failure in the specimen.

## 1. Introduction

Carbon anodes are part of the chemical reaction of the alumina reduction and they are consumed during the Hall-Héroult electrolysis process. The mechanical and chemical qualities of the carbon anodes directly affect the technological, economical, and environmental aspects of the aluminum production process. The carbon anode production accounts for 17% of the total cost of the aluminum smelting [1]. In addition, to produce one ton of aluminum, theoretically, 334 kg of carbon would be required. However, in practice, the required carbon is higher and roughly about 415 kg per ton of aluminum [2]. Hence, improving the chemical and mechanical properties of the anode not only reduces the cost of aluminum production, but it also reduces the environmental impact of aluminum production by consuming less carbon and, thus, producing fewer greenhouse gases.

The carbon anodes are composed of three major parts, i.e. the calcined petroleum coke (65 wt.%), the recycled anode (butt, 20 wt.%), and the coal tar pitch (15 wt.%). Initially, the coke particles are crushed and sieved to the required size distribution, and they are mixed with the granulated recycled butts. The dry aggregates are then heated to about 160 ${}^{{}^{\circ}}$C and mixed with the coal tar pitch at 150–180 ${}^{{}^{\circ}}$C. The coal tar pitch binds the coke and butt particles. The obtained mixture is called the anode paste. The anode paste goes through the vibro-compaction or the pressing process to form the green anode blocks. The green anodes are baked at a temperature of 1100 ${}^{{}^{\circ}}$C to improve the mechanical strength and electrical conductivity. Subsequently, the obtained baked anodes can be used as electrodes in the aluminum smelters [3].

High mechanical strength and electrical conductivity, homogeneity, as well as low reactivity towards carbon dioxide and air are the important quality indices of the carbon anodes [4]. The main parameters determining the final anode quality are categorized into two essential groups; the material properties and the process parameters [5]. One of the most significant challenges in the anode manufacturing industry is that its raw materials do not always have the same properties. This quality variation is due to the fact that the raw materials come from different sources. When the material properties are changed, the paste formation and process parameters, including the mixing variables and the compaction parameters, should be re-adjusted in such a way to compensate for the effects of the variations and to keep the anode quality consistent. Moreover, the sufficient mixing power and time, the optimized speed of the vibro-compaction, and the confining pressures, as well as the proper temperature, are the most important process parameters determining the mixing effectiveness and the anode quality. An efficient mixing results in a homogeneous distribution of the coke and the coal tar pitch, and lower porosity in the paste that improves the anode characteristics, such as the density and thermal shock resistance [4]. In addition, any changes in either the speed and load of pressing forming or the frequency and dead-weight of the vibro-compaction process influence the homogeneity of the density of the green anodes, as well as the quality of the baked anodes [6]. Similarly, the higher baking temperature leads to larger crystallite sizes and a more homogeneous structure of the pitch-coke, which reduces the electrical resistivity and consumption rate of the carbon anodes [7].

**Figure 1:**
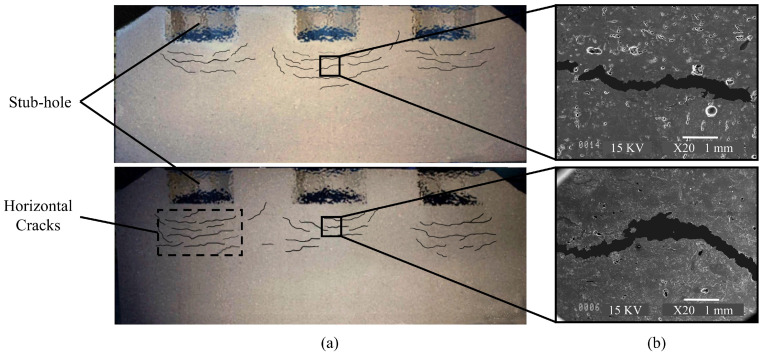
(**a**) Images of the horizontal cracks under the carbon holes of the anodes, which were obtained after cutting the anodes made in the Alcoa Deschambault Québec (ADC) (The size of the cracks has been magnified for greater clarity), (**b**) Images of the cracked area by Scanning Electron Microscope (SEM).

Any defects, such as the internal and the external cracks and the density distribution, affect the carbon anode consumption rate and remarkably increase the process costs [6]. The presence of the cracks reduces the mechanical strength and the electrical conductivity of the baked carbon anode, thereby reducing the life of the carbon anode, disrupting the cell stability, and increasing the greenhouse gas emissions [5]. Given that all of the steps of anode production are done at high temperatures and the components of the anode paste are opaque, it is not easy to investigate the origins of the cracks. Three major types of cracks can develop in the carbon anodes: corner, vertical, and horizontal cracks [8]. The corner cracks predominantly appear after the anode is set into the electrolysis cell due to the thermal shock [9]. The vertical cracks are mainly created during the baking process. The high temperature gradient inside the carbon anode due to the high heating rate provides the tensile stresses that are required to create the vertical cracks [7]. The horizontal cracks of the anodes are the most detrimental to the electrolysis operation [8]. Under normal circumstances, the stresses that are caused by the thermal shock cannot generate these types of cracks [8]. These defects should already appear as small horizontal cracks that are likely to occur during the formation process [8]. Boubaker et al. [10] reported a kind of the horizontal cracks below the stub-holes of the baked carbon anodes. In Figure 1, the baked carbon anodes are cut from the middle and shows the horizontal cracks under the stub-holes. Although these cracks are not present in all the anodes, they are accidentally observed beneath the stub-holes. However, in the compaction process, the compression stresses around the stub-holes appear to be higher than in other parts of the carbon anode. Hence, It seems strange to have these types of cracks where they are probably denser than elsewhere in the anode [11]. On the other hand, because these cracks are the opening type, the tensile stresses perpendicular to the direction of the crack growth is required to generate them [12]. However, the origin of these tensile stresses beneath the stub-hole is not known [10].

Many investigations have been conducted to find the cause of the formation of the cracks [11,13].The experimental investigations are not easily performed because of the high temperature of the forming process and the opacity of the carbon anode paste. Chaouki et al. [11] proposed a constitutive law to simulate the anode paste during the compaction process. Although this model can reveal the density gradient due to the stub-hole, it is not capable of demonstrating the formation of the horizontal cracks below the stub-hole [11,13]. This limitation stems from the fact that the granularity nature of the anode paste cannot be taken into account by phenomenological models such as finite element methods [14]. On the other hand, several attempts have been made to investigate the behavior of anode paste using the discrete element method (DEM), which considers grains as the basic element from which the mechanical behavior of granular materials originates [5,15]. Despite the fact that modeling anode paste with all its complexities, including different size distribution, particle shape, solid-fluid interaction, and coal-tar pitch dependence on temperature, is a challenging task, DEM has shown that it is able to successfully simulate some properties of the anode coke aggregates, such as the bulk density [16] and the electrical resistivity [17]. However, investigating the causes of the horizontal cracks under stub-holes requires more in-depth analysis. Hence, a comprehensive study of the distinct behaviors of granular materials that are subjected to compression loading conditions can shed light on the hidden truth of this problem.

The granular materials are generally defined as materials that consist of the smaller particles and their mechanical behavior is governed by the interaction between their particles [14]. When the granular material is exposed to a compression load, it reaches a stress state wherein it is no longer able to sustain any deviatoric load increment. At such a limited condition, if even a small additional load is applied to the material, it will suddenly undergo the occurrence of large deformations, cracks, fragmentation, etc. [18]. This circumstance, which is associated with a sudden decrease in the number of grain contacts, is called failure [19]. The sudden reduction in the grain contacts will be accompanied by a significant increase in the number of degrees of freedom. This means that the probability of rapid relative displacements between the particles increases. Accordingly, the failure can be interpreted as a physical phenomenon, in which a quasi-static regime can be transformed into a dynamical regime while the loading parameters remain constant [20]. For the materials with an associative flow rule, as it is generally assumed for metals, the symmetry of the elasto-plastic tensor leads to the compelling fact that the failure occurs in the plastic limit condition. However, for granular materials, which are known to have non-associated flow rules and, consequently, non-symmetry in the elasto-plastic tensor, the failure can be met before the plastic limit condition (Mohr–Coulomb criterion) [21]. The mathematical interpretation of the failure is usually attributed to the existence of a limit load that cannot be exceeded for a given mechanical system under some boundary and initial conditions [22].

The failure in the granular materials is initiated by the instability of these materials [23]. The instability can be either geometric, such as structural instability [24], or material, such as constitutive behavior and force chains buckling [21,25]. The geometric instability is associated with the tendency of the configuration to pass from one deformation pattern to another [24]. For instance, the critical condition of a long, slender column that is axially loaded is a state of transition from pure compression to a combination of compression and bending. Therefore, this type of instability is a function of the geometry of the specimen and its loading [26]. On the other hand, the material instability is defined as a property of the material that converts an initially homogeneous deformation field into a heterogeneous deformation field [27]. The material instability is related to the size of the materially intrinsic length scales, which is called microstructure, and the magnitudes of the length scale of the initial perturbations [25,27]. For example, local buckling of particle force chains is considered to be a material instability [25,28].

The material instability causes the underlying governing equation not to have a unique solution, thus it will become a bifurcation problem [29]. When a mechanical state pertains to the bifurcation domain, the possibility of failure in addition to the loading parameters, loading history, and imperfection in the system, is strongly dependent on small disturbances [18]. Hence, the dependence of failure on small perturbations makes it possible to consider it as a phenomenon of instability in the original sense of Lyapunov [30]. The Lyapunov definition of stability expresses that, for a given rate-independent material, a stress–strain state for a given strain history is called stable if any small change in any acceptable loading results in a slight change in the response. However, the main question that comes to mind is, according to Lyapunov’s definition of stability, how can be shown a stress-strain state is unstable strictly inside the plastic limit surface?

Two concepts of failure are built around the above-mentioned question of describing the failure. The first one is the notion of controllability [31] and the second one is the sustainability of equilibrium states [32]. Nova [31] defines the controllability as the ability of a material (or a model) to supply one, and only one, response (uniqueness and existence) under any given loading path. Accordingly, the granular materials lose their controllability at a certain stress level and, after that point, they do not give rise to a unique material response under any arbitrary incremental loading program. At this point, the stiffness tensor is no longer positive definite. It has been shown that, as soon as the stiffness tensor becomes positive semi-definite, there is a particular program that leads to infinite solutions and unconditional controllability is lost [33]. Therefore, as the notion of controllability applies to a given loading program, this is not an intrinsic characteristic of the mechanical state of the system [31]. On the other hand, another interpretation of the Lyapunov definition of stability is regarding the sustainability of the mechanical state of the system. In this interpretation, if a loaded mechanical system can be converted from a given equilibrium state to another mechanical state, while the control parameters are fixed, the equilibrium state of the material will not be sustainable; consequently, the state of the mechanical system belongs to a bifurcation domain [32]. From a mechanical point of view, it means that a system that is initially in equilibrium can generate kinetic energy spontaneously and without any external disturbances [22,32].

Because of the difficulty with Lyapunov definition of stability, there was a need for a related manageable criterion of failure for the practical use in the investigation of the granular materials [34,35]. To compensate for this issue, Hill’s second-order work criterion of stability has been introduced. Hill’s criterion [36] states that a stress-strain state is stable if it can maintain its state against infinitesimal disturbances in the absence of an external energy source. Although Hill’s criterion and Lyapunov’s definition of stability are not related in a general manner [37], the concepts of controllability and sustainability are equivalent to the Hill’s criterion in the classical elasto-plasticity [31] and the failure of granular materials [35,38]. Therefore, in spite of the fact that this criterion does not specify the mode of material failure [20], it can predict the necessary conditions for the occurrence of a failure in the granular materials.

Various modes of failure in granular materials have been observed in practice. Thanks to experimental observations, there are two broad classes of failure modes that arise in the granular materials due to some instabilities [39]. In the granular materials, excluding flutter instabilities, two material failure modes are of interest: localized and diffuse failure modes. The localized failure is a mode of failure in which the strain pattern of a material change from homogeneous to heterogeneous, being characterized by the emergence of a system of bands in which the strain is concentrated [20,40]. These narrow zones where deformation is concentrated are called localized bands. Shear, dilation, or compaction bands may be developed, depending on the loading path and their kinematic attributes [41]. While the shear bands are predominated by shearing, the dilation and compaction bands are primarily formed by volumetric deformation and they are characterized by local volume expansion and local volume reduction, respectively [41]. The strain localization of the granular materials has been studied by many researchers through theoretical [40,42], experimental [43,44,45,46,47,48], and numerical methods [49,50,51,52]. There have been attempts to simulate the phenomenon of the strain localization in the granular material, especially in the sand samples, based on either continuum mechanics by using the finite element method (FEM) [49,50] or micro-mechanics by using the discrete element methods (DEM) [47,51]. The finite element methods (FEM) require the constitutive relation of the material, while there are no reliable constitutive laws that can accurately predict the behavior of the granular materials [53]. It should be noted that the constitutive laws that are derived from the classical continuum mechanics do not take into account the dimensions of the granular elements [14,54]. Consequently, these constitutive laws suffer from pathological mesh-dependency when they are employed in the failure analyses [55,56]. However, the discrete element method can provide applicable equipment for considering the internal length scale of the granular material without involving the sophisticated mathematics of the non-classical continuum mechanics [57]. In addition, a combination of the latter two methods, called multi-scale methods, is also used to model the strain localization in the granular materials, which benefits from both FEM and DEM [14,52,56,58,59].

On the contrary, the diffusing failure mode corresponds to a homogeneous occurrence of the failure in which no visible pattern of localization exists [60]. A chaotic, unstructured strain field dominates [37]. This failure mode can mostly be observed in the loose sand specimens for classical tests [61]. Diffusing failure does not occur in the dense sand under undrained conditions [62]. For instance, an isochoric triaxial test performed on a loose sand specimen showed that applying an infinitesimal loading disturbance to the sample, when it is at the peak of deviatoric stress, causes a collapse of the specimen without any specific pattern of localization [34,60]. While the localized failure is predicted by the vanishing values of the determinant of the acoustic tensor [42], which is known as classical bifurcation analysis, the second-order work criterion is mostly used as a proper indicator of the diffuse failure mode [60]. Although there are differences in the kinematics properties of the two failure modes, Ref. [63] showed that both localized and diffuse failure can be predicted through the classical bifurcation analysis. Despite the difficulty in finding a proper constitutive law that describes the granular material’s behavior, the bifurcation analysis has been used widely to predict failure in the sands [50,64], the rocks [65], and the fluid-saturated granular soils [66,67]. Moreover, it has been shown that the second-order work criterion is capable of detecting both the diffuse and localized failure modes [20]. This criterion, unlike the classical bifurcation analysis, does not necessarily require a constitutive law to predict failure [68].

Comprehension of failure as a mechanism to generate defects in granular material can reveal the facts about the origin of the imperfections such as cracks in the granular materials (e.g., see [41,69] and the references cited in them). In geology, the localized bands are recognized as the main mechanism of fault formation in sandstone that precedes the formation of the larger faults [70,71]. Because these localized bands are usually associated with porosity reduction, they may provide a natural barrier to fluid flows and form hydrocarbon reservoirs and aquifers [72,73]. Another type of localized bands, called compaction bands, is formed by the accommodation of pure compaction (with little or no shear) in the tabular zone perpendicular to the maximum compression direction in the sandstone or the sedimentary rocks [74,75,76]. There are compelling evidence for the existence and the formation of compaction bands in the granular materials that are exposed to the compressive stress states both in the laboratory and in the theory [76]. Although compaction bands were first recognized in the sandstone [74], similar phenomena appear to be common in the other porous materials [77]. For instance, Bastawros et al. [78] were able to illustrate the formation of the compaction bands in a cellular aluminum alloy upon axial compression through a digital image correlation procedure. Similar observations had been reported for steel foams [79] and polycarbonate honeycombs [80], in which inherent pore collapse has mainly caused the formation of the compaction bands.

**Figure 2:**
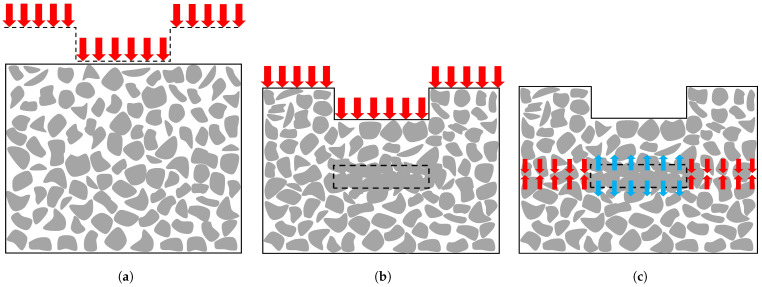
Generation of the residual tensile stresses due to compaction band formation. (**a**) The carbon anode paste before the compaction process. (**b**) The carbon anode paste during the compaction process and the formation of the compaction band (dashed rectangle). (**c**) Creating residual stresses in the absence of the external pressure. (The red arrows indicate the compression stresses and the blue ones show the tensile stresses).

The characteristics of the compaction bands, such as being perpendicular to the maximum principal compression direction, as well as the similarity in the way of loading, which is mainly compressive, have led us to the idea that these bands can generate the horizontal cracks beneath the stub-holes in the carbon anodes. Figure 2 shows how internal tensile stresses could generate inside the carbon anode, even in the absence of an external load. When the compressive stresses are applied to the carbon anode paste, due to the stub-hole shape effect, the areas below the stub-holes subject to more compaction than their neighboring areas (Figure 2a). It is assumed that this additional compression can cause the compressive strain to accumulate in a narrow rectangular region, resulting in a compression band (dashed rectangle in Figure 2b). After removing the external load from the material, due to the viscoelastic properties of the carbon anode paste, the compression that accumulated in the compaction bands causes residual tensile stresses in the stub-hole region, as well as residual compressive stresses in the neighboring areas (Figure 2c). Accordingly, the compaction bands could be responsible for the tensile stresses that are required for the generation of these type of cracks. This phenomenon is similar to the inclusion problem in the elastic media described by Eshelby [81]. Although many researchers used an analogous method to predict the initiating of the compaction bands in the porous rocks [75,82,83,84], the factors influencing the various manifestations of the compression bands are still unknown [76]. Therefore, understanding the failure behavior of the granular materials is of great importance for finding the mysterious phenomena of compaction band formation. In addition, due to the fact that detection of the compaction bands is difficult in either the field or the laboratory [76], it is possible that compaction bands are present in virtually all of the carbon anodes (even in the cases where there are no horizontal cracks). Although some parameters, such as thermal shocks or shrinkage of the coal-tar pitch during the baking process, affect the formation of the cracks in the carbon anodes, the compaction bands are a mechanism that can create a susceptible region under the stub-holes to generate the horizontal cracks. Therefore, it is necessary to determine the factors of the physical conditions and the material characteristics that are associated with the formation of the compaction bands in the case of a systematic investigation.

**Figure 3:**
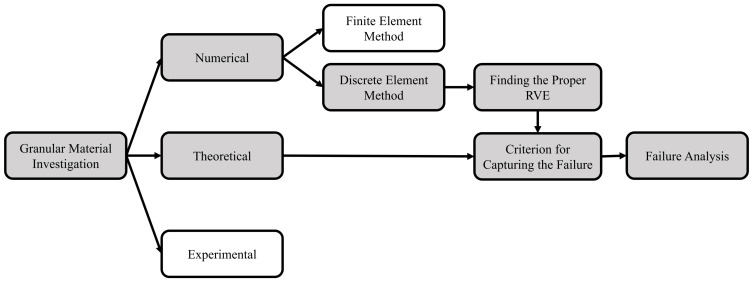
The flowchart of coke aggregate failure analysis approaches (the gray boxes outline the selected strategy in this paper).

As aforementioned, the existence of compaction bands in the visco-elastic anode paste creates a susceptible area for the horizontal crack formation. While the temperature and coal-tar pitch content affect the viscous part of the anode paste, the coke particle characteristics influence the elastic part of the anode paste behavior [4]. Therefore, it seems reasonable to only consider the coke particles for the failure analysis. In addition, the coarse coke particles have been shown to form a skeleton that controls the main mechanical behavior of coke aggregates [85]. Hence, for the sake of simplicity, we will consider the coarse coke particles with spherical shape for our investigations. Figure 3 manifests the strategy chosen for the coke aggregate failure analysis in this paper. Accordingly, the second-order work criterion for the failure of the granular material will be reviewed and the influence of failure on the kinetic energy of the system will be explained in Section 2. In addition, the ability of the second-order work criterion in diagnosing the failure of the granular material will be discussed. In Section 3, the concept of the discrete element method will be presented. The criteria for choosing the proper representative volume element (RVE) will be studied. In Section 4, the strain localization analysis is presented based on the second-order work criterion and the evolution of the mode of the localized bands will be discussed. Section 5 willl summarize and discuss the most salient results of this work.

Throughout this paper, the material time derivatives of any variable ψ will be distinguished by denoting $\frac{D\psi }{Dt}$ and the particulate time derivative of ψ defined as $\dot{\psi }$. The first-order tensors (vectors) and the second-order tensors, respectively, denoted by lower-case bold Latin (v) and upper-case bold Latin (F), while the italic form of Latin letters indicates the components of the tensors. In addition, the scalar product of two first-order tensors (vectors), v and u, and the double contraction of two second-order tensors, S and R, are indicated by (v·u) and (S:R), respectively. Moreover, the subscript 3 throughout the paper indicates the axial direction, while the subscripts 1 and 2 were designated as lateral directions.

## 2. Second-Order Work Criterion

In mechanical systems for which a potential energy function can be defined, the stability of the system is guaranteed if this potential function has a strict minimum. Because of the complex physical phenomena and dissipation mechanism, there is no potential energy function in the mechanical problems that are associated with the granular media [34]. Therefore, the material instabilities in the granular materials cannot be investigated through the potential energy function analysis. In other words, these instabilities are linked to the inherent deformation mechanisms of the granular material and they do not depend on the potential energy. In addition, the theoretical investigations, the numerical analyses, and the experimental results highlight that the concept of failure is related to the development of kinetic energy [22,62,86,87]. As a consequence, it is necessary to have criteria that relate the kinetic energy of granular material to the control parameters (such as strain or stress at the boundaries). Hence, the issue of stability will be investigated using Hill’s second-order work criterion [36]. This sufficient condition of failure states that a stress–strain state is stable if, for all (δP,δF) in the semi-Lagrangian formulation or (δσ, δϵ) in Eulerian formulation (by assuming small deformations and neglecting geometrical aspects) linked by the constitutive relation, the second-order work is strictly positive [88]:

(1) $$\begin{array}{cc} d^{2}W= & \underset{V_{0}}{\;\int \int \int \;}\delta P_{ij}\;\delta F_{ij}\;dV_{0}>0\;(\mathrm{semi}-\mathrm{Lagrangian}\;\mathrm{expression})\;,\\ d^{2}W= & \underset{V}{\;\int \int \int \;}\delta \sigma_{ij}\;\delta \epsilon_{ij}\;dV>0\;(\mathrm{Eulerian}\;\mathrm{expression})\;, \end{array}$$

where $P_{ij}$ is the first Piola–Kirchhoff stress tensor, $F_{ij}$ the general term of the deformation gradient tensor, $\sigma_{ij}$ the Cauchy stress tensor, and $\epsilon_{ij}$ is the strain tensor. Hence, according to Hill, a stress–strain state will be unstable when there is at least one loading direction that can be converted to another state in an infinitesimal manner without any external energy [89]. In fact, Hill’s sufficient condition of stability states that vanishing of the second-order work, regardless of the type of material constitutive relations, can lead to a loss of controllability of the loading program [33]. Although this sufficient condition does not originate from thermodynamic principles, it is known as a valuable tool for describing any type of quasi-static material instability, especially for granular materials, because its use does not necessarily require the constitutive relationships of the materials [34].

### Kinetic Energy of the Granular System and External and Internal Second-Order Work

**Figure 4:**
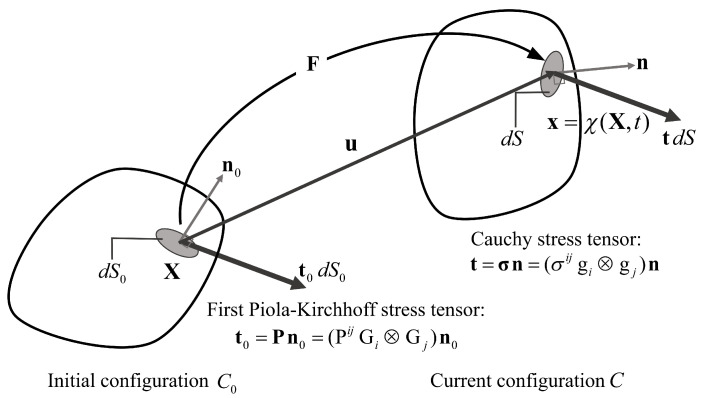
Definition of the First Piola–Kirchhoff stress tensor and Cauchy stress tensor and transformation of a material system.

An attempt for the definition of the failure in the granular material was made in the previous section, and this related to a transition (bifurcation) from a quasi-static regime toward a dynamic one. In this section, the mathematical description of the second-order work criterion is developed and the conditions in which the kinetic energy of the granular material system may increase will be investigated. For this purpose, a system consisting of granular material, with a volume, $V_{0}$, and a surface boundary, $S_{0}$, initially in a configuration, $C_{0}$, is considered. With a loading history, the system is in a current configuration, *C*, with volume, *V*, and the surface boundary, *S*, in equilibrium under a prescribed external load. Each material point in the volume $V_{0}$ is transformed into a material point in the volume *V* (Figure 4). All of the material points in the volume $V_{0}$ are displaced along with the deformation of their geometric properties, including the surface vector, the area, and the volume. During this transformation, the material is subjected to a rigid body motion, along with the pure strain that is induced by the stretching and the spinning deformations. If large amounts of strain take place, the initial configuration, $C_{0}$, will be significantly different from the current configuration, *C*.

Because the Cauchy stress tensor is not objective (in the rigid body transformation, it gives different values), the first Piola–Kirchhoff stress tensor and the conservation of the mechanical energy in the material description are used [90,91]. It should be noted that the first Piola–Kirchhoff stress vector is the vector $t_{0}\left(X,t,n_{0}\right)$, which is parallel to the Cauchy stress t(x,t,n), but it measures the force per unit undeformed area (see Figure 4). The balance of the kinetic energy of a system with neglecting the body force in the material description (configuration $C_{0}$) can be derived as [92]:

(2) $$\frac{D}{Dt}K\left(t\right)=P_{ext}\left(t\right)-P_{int}\left(t\right)\;,$$

or

(3) $$\frac{D}{Dt}\underset{V_{0}}{\;\int \int \int \;}\left(\frac{1}{2}\rho_{0}v\cdot v\right)dV_{0}=\underset{S_{0}}{\;\int \int \;}Pn_{0}\cdot vdS_{0}-\underset{V_{0}}{\;\int \int \int \;}P:\dot{F}dV_{0}\;.$$

Equation (3) expresses that the rate of change of the kinetic energy, K(t), is equal to the difference between the power of the external forces, $P_{ext}\left(t\right)$, and the power of the stresses, $P_{int}\left(t\right)$. The stress power, $P:\dot{F}$, given in term of the first Piola–Kirchhoff stress tensor $P=J\sigma F^{-T}$ and the deformation gradient F. Note that the stress power $P:\dot{F}$ refers to the unit undeformed volume. By taking the time derivative of Equation (3) yields:

(4) $$\frac{D^{2}}{Dt^{2}}\underset{V_{0}}{\;\int \int \int \;}\left(\frac{1}{2}\rho_{0}v\cdot v\right)dV_{0}=\underset{S_{0}}{\;\int \int \;}\left(\dot{P}n_{0}\cdot v+Pn_{0}\cdot \dot{v}\right)dS_{0}-\underset{V_{0}}{\;\int \int \int \;}\left(\dot{P}:\dot{F}+P:\ddot{F}\right)dV_{0}\;.$$

Furthermore, the two-order Taylor expansion of the kinetic energy reads:

(5) $$K\left(t_{0}+\Delta t\right)=K\left(t_{0}\right)+\Delta t\dot{K}\left(t_{0}\right)+\frac{{\left(\Delta t\right)}^{2}}{2}\ddot{K}\left(t_{0}\right)+H.O.T.\;\left(\Delta t\right)\;.$$

Because the velocity of the system in the initial time is equal to zero (quasi-static), the amount of the kinetic energy $K(t_{0})$ and its first time derivative $\dot{K}\left(t_{0}\right)$ must be equal to zero [87]. In addition, if Δt is considered to be small, then the higher-order terms of Δt (H.O.T. (Δt)) can be ignored. Therefore, by substituting in Equation (5), the kinetic energy in a very small time interval could be predicted as:

(6) $$K\left(t_{0}+\Delta t\right)=\frac{{\left(\Delta t\right)}^{2}}{2}\ddot{K}\left(t_{0}\right)\;.$$

Therefore, by combining Equations (4) and (6), an approximation of the kinetic energy changes in a quasi-static system will be obtained as a function of the external and the internal stress powers.

(7) $$K\left(t_{0}+\Delta t\right)=\frac{{\left(\Delta t\right)}^{2}}{2}\left(\overset{{\dot{P}}_{ext}\left(t\right)}{\overset{︷}{\underset{S_{0}}{\;\int \int \;}\left(\dot{P}n_{0}\cdot v+Pn_{0}\cdot \dot{v}\right)dS_{0}}}-\overset{{\dot{P}}_{int}\left(t\right)}{\overset{︷}{\underset{V_{0}}{\;\int \int \int \;}\left(\dot{P}:\dot{F}+P:\ddot{F}\right)dV_{0}}}\right)\;.$$

Based on Equation (7), the evolution of the kinetic energy of a granular system for every time step can be expressed as the difference between the rate of the external and the internal stress power. It should be noted that this approximation is only valid for small time intervals. In addition, in Equation (7), it is important to distinguish the external stresses that were applied to the boundary and the stresses inside the boundary.

**Figure 5:**
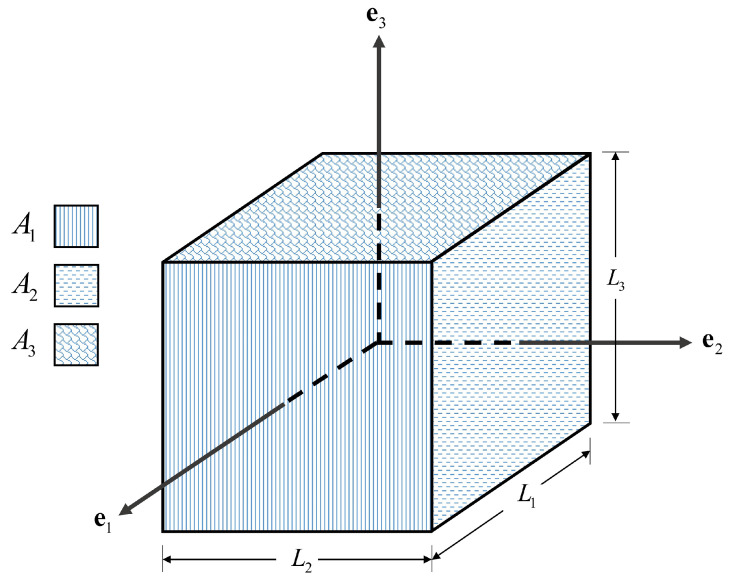
Cubic representative volume element.

Some simplification needs to take place for using Equation (7). Hereafter, we particularize the analysis to a cubic representative volume element with dimension $\left(L_{1}\times L_{2}\times L_{3}\right)$ as defined in Figure 5. The average external stress at the boundaries is determined by the sum of the contact forces along the boundary, f, divided by the surface area of the rigid boundary, $A_{i}$. Therefore, the average external stress of each side of the boundary for a 3D model is equal to:

(8) $$T_{i}=\frac{f_{i}}{A_{i}}\;,$$

where, $f_{i}$ is the equivalent external force on the side “*i*” and the $A_{i}$ is the area of the surface perpendicular to the direction “$e_{i}$”, as mentioned in Figure 5. The displacement of each side is denoted $u_{i}=u\cdot e_{i}$. The deformation gradient tensor is defined as $\left[F_{ij}\right]=\frac{\partial x_{i}}{\partial X_{j}}=1+\frac{\partial u_{i}}{\partial X_{j}}$. No tangential displacement is assumed to take place. Therefore, the deformation gradient tensor will be in its principal axes. It should be noted that at any material point of the system, both the rate of the first Piola–Kirchhoff stress tensor ($\dot{P}$) and the rate of the deformation gradient tensor ($\dot{F}$) are related by the constitutive equation ${\dot{P}}_{ij}=L_{ijkl}{\dot{F}}_{ij}$, where the four-order tensor L is the tangent constitutive tensor for rate-independent materials. Because the first Piola–Kirchhoff stress tensor and deformation gradient tensor are each other’s energy conjugate, the first Piola–Kirchhoff stress tensor will be, in principle, axes as well. Against this background, it could be written:

(9) $$\langle F\rangle =\left[\begin{array}{ccc} \langle F_{11}\rangle & 0 & 0\\ 0 & \langle F_{22}\rangle & 0\\ 0 & 0 & \langle F_{33}\rangle \end{array}\right]\;\mathrm{and}\;\langle P\rangle =\left[\begin{array}{ccc} \langle P_{11}\rangle & 0 & 0\\ 0 & \langle P_{22}\rangle & 0\\ 0 & 0 & \langle P_{33}\rangle \end{array}\right],$$

where, $\langle Y\rangle$ denotes the mean value of the variable *Y* over the whole volume $V_{0}$, which is defined as:

(10) $$\langle Y\rangle =\frac{1}{V_{0}}\underset{V_{0}}{\;\int \int \int \;}YdV_{0}\;.$$

For the deformation gradient tensor $\langle F_{ii}\rangle =\frac{1}{V_{0}}\underset{V_{0}}{\;\int \int \int \;}\left(1+\frac{\partial u_{i}}{\partial X_{j}}\right)dV_{0}$ by virtue of the Green formula, the following hold:

(11) $$\langle F_{ii}\rangle =\frac{1}{V_{0}}\left(\underset{V_{0}}{\;\int \int \int \;}dV_{0}+\underset{S_{0}}{\;\int \int \;}u_{i}e_{i}dS_{0}\right)=1+\frac{A_{i}}{V_{0}}u_{i}\;.$$

The detailed mathematical calculations of the first and the second rate of the deformation gradient tensor are provided in Appendices Appendix A and Appendix B, respectively.

By considering the rate of the external stress power, ${\dot{P}}_{ext}\left(t\right)$, and the above assumptions, it could be simplified as:

(12) $${\dot{P}}_{ext}\left(t\right)=\underset{S_{0}}{\;\int \int \;}\left[\left(\frac{\partial T_{i}}{\partial t}\right)\left(\frac{\partial u_{i}}{\partial t}\right)+T_{i}\left(\frac{\partial^{2}u_{i}}{\partial t^{2}}\right)\right]dS_{0}\;.$$

Equation (12) can be written as:

(13) $${\dot{P}}_{ext}\left(t\right)=\sum_{i=1}^{3}\left({\dot{T}}_{i}{\dot{u}}_{i}+T_{i}{\ddot{u}}_{i}\right)A_{i}\;.$$

due to considering a fixed value of the external stress on each side of the boundary.

On the other hand, the macro-homogeneity assumption makes it possible to invoke the fundamental Hill identity [87], stating that $\langle P_{ij}F_{ij}\rangle =\langle P_{ij}\rangle \langle F_{ij}\rangle$, consequently, by considering the mean value for the first Piola-Kirchhoff stress tensor and the deformation gradient tensor, the rate of the internal stress power, ${\dot{P}}_{int}\left(t\right)$, could be written as:

(14) $${\dot{P}}_{int}\left(t\right)=\underset{V_{0}}{\;\int \int \int \;}\left(\langle {\dot{P}}_{ij}\rangle \langle {\dot{F}}_{ij}\rangle +\langle P_{ij}\rangle \langle {\ddot{F}}_{ij}\rangle \right)dV_{0}\;.$$

Combining Equations (11) and (14) gives:

(15) $${\dot{P}}_{int}\left(t\right)=\left(\langle {\dot{P}}_{ij}\rangle \langle {\dot{F}}_{ij}\rangle +\langle P_{ij}\rangle \langle {\ddot{F}}_{ij}\rangle \right)V_{0}=\sum_{i=1}^{3}\left(\langle {\dot{P}}_{ii}\rangle {\dot{u}}_{i}+\langle P_{ii}\rangle {\ddot{u}}_{i}\right)A_{i}\;.$$

By substituting Equations (13) and (15) in Equation (7), an expression of the kinetic energy as a function of the system’s second-order works is obtained:

(16) $$K\left(t_{0}+\Delta t\right)=\frac{{\left(\Delta t\right)}^{2}}{2}\sum_{i=1}^{3}\left[\left({\dot{T}}_{i}-\langle {\dot{P}}_{ii}\rangle \right){\dot{u}}_{i}+\left(T_{i}-\langle P_{ii}\rangle \right){\ddot{u}}_{i}\right]A_{i}\;.$$

The first term of the right-hand side of Equation (16) represents the difference between external and internal second-order work. The second term ($\left(T_{i}-\langle P_{ii}\rangle \right){\ddot{u}}_{i}A_{i}$) demonstrates the effect of the inertia on the evolution of the kinetic energy. According to Equation (16), the external stress vector ($T_{i}$) acting on the boundary of the specimen is equal to the internal stress ($\langle P_{ii}\rangle$) acting within the specimen as long as the system is in the quasi-static evolution. As a result, the measurable variables $T_{i}$ and $u_{i}$ at the boundary can be considered to be the constitutive response of the specimen. These variables are exactly the same information that can be obtained from the laboratory experiments. Therefore, it can be inferred that the laboratory data will reveal the constitutive response of the specimen as long as the system is in a quasi-static state. On the other hand, when the material failure occurs, the transition from the quasi-static to the dynamic regime, the information obtained from the boundary is not the exact information of the material constitutive relations. In this case, the specimen may undergo a heterogeneous deformation field due to the fact that the external stresses are not being balanced by the internal stresses [87]. In addition, when the failure occurs, the internal stress will be dropped and according to Equation (16), the terms $\left({\dot{T}}_{i}-\langle {\dot{P}}_{ii}\rangle \right)\;\mathrm{and}\;\left(T_{i}-\langle P_{ii}\rangle \right)$ are greater than zero. Therefore, it leads to $K(t_{0}+\Delta t)>0$, which describes an outburst in the kinetic energy [86]. Hence, a sudden release in the kinetic energy of the system, could be an indicator of the material failure.

## 3. Discrete Element Method (DEM) Simulation

The discontinuous nature of the granular materials causes many phenomena, such as the collapse of void space and the buckling of force chains, which cannot be modeled by the phenomenological plasticity methods [57,58]. One possibility to obtain information about the behavior of the granular materials is to perform simulations with the discrete element method (DEM), as proposed by [93]. Because the DEM provides the opportunity to track the motion of every single particle in the grain assembly, it can consider how the microstructures affect the macroscopic properties of the granular material. In fact, what makes the discrete element methods popular is the ability to describe the physics and mechanics of granular materials whose behavior is influenced by their smaller components. While it would be difficult to investigate the effects of these smaller components experimentally. Therefore, it provides interesting information to describe the mechanisms of the failure in the granular materials.

**Figure 6:**
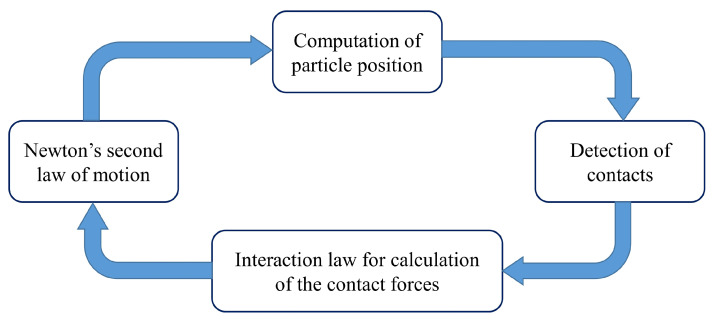
The computation cycle of a discrete element method (DEM) modeling.

In this paper, the DEM computations were realized with the open-source software YADE [94]. The particles are assumed to be rigid spheres with a diameter, $d_{p}$. The use of spherical particles increases the simulation efficiency. For instance, it simplifies the collision detection calculations. In this case, the collision between two particles occurs when the distance between the centers of the two particles is less than the sum of their radii. The interactions between the particles are simulated in the normal direction to the contact by a linear elastic spring with a stiffness $K_{n}=681$ MPa, and in the tangential direction by a linear elastic spring with a stiffness ($K_{t}/K_{n}=0.385$), and the tangential perfect plasticity with a friction angle $\varphi =18^{{}^{\circ}}$ [5]. The normal and the tangential contact forces, $f_{n}$ and $f_{t}$, respectively, are given by [93]:

(17) $$\begin{array}{cc} f_{n}= & K_{n}\delta_{n}\;f_{n}>0\;,\\ f_{t}= & K_{t}\delta_{t},\;f_{t}⩽\tan\varphi f_{n}\;, \end{array}$$

where $\delta_{n}$ is the overlap at the contact point and $\delta_{t}$ is the incremental tangential displacement. At the beginning of a computational time-step, the position of all the elements and boundaries are known. The contacts are detected by the algorithm according to the known position of the elements and so the magnitude of the possible overlaps between the elements are discovered. The propagated contact forces and momentum on each sphere are then calculated by the interaction law (Equation (17)). After that, the forces are inserted in Newton’s second law of motion for each particle and the velocity and the acceleration of the particles are calculated. Subsequently, the new positions of the spheres are calculated by applying Newton’s second law of motion. The explicit integration method is used to implement both Newton’s second law and the interaction contact law. The positions of all the particles and the boundaries in the current time-step are determined by the obtained values. This cycle of the calculations is repeated and solved at each time-step and, thus, the flow or the deformation of the material is simulated (Figure 6).

The simulation results presented in this paper were all obtained from two boundary conditions, the periodic and the solid boundary conditions. In the periodic boundary conditions, the particles can go through the boundaries, although the total number of the particles is constant. It is useful for the bulk properties modeling, because it ignores the boundary effect on the behavior of the material [95]. Meanwhile, the solid boundary conditions are used for the failure analysis, which is strictly controlled by the boundary effects [96]. Here, it is assumed that the solid boundaries are frictionless. Therefore, the interaction of the spheres and the walls will be in the normal direction of their contacts. The specimens are generated by randomly inserting grains within a cubic domain (each side is $D_{initial}=8$ cm long) with the possibility of overlap until a target void ratio is achieved. Afterwards, specimens are left to stabilize. Because the time required to complete the calculation depends on the number of particles, determining the optimum number of particles is a challenging part of our work.

### 3.1. Determination of a Proper Representative Volume Element (RVE)

The modeling of the real size of the carbon anode is not practical because of the high computational cost of the DEM simulation. Therefore, we need to perform our simulation on the optimum number of particles, which could represent the mechanical behavior of the whole material with an acceptable statistical error [97]. Accordingly, six different representative volume elements (RVE) are considered, each of which contains 150, 300, 500, 1000, 2000, 3000, and 4000 particles, respectively. The particle diameter is the same and is equal to 3.74 mm. This is the average diameter of the coarse coke (4–8 US mesh) [15]. Table 1 also provides the properties of the materials. All of the RVEs are then consolidated to the same initial confining pressure $P_{0}=100$. Because of the mechanical properties of the RVEs are intended here, their shear responses are examined under a drained conventional triaxial compression loading path. Hence, the load is applied through the displacement-controlled boundaries in the z-direction ($\dot{\epsilon_{3}}=0.05\;s^{-1}$), while the lateral boundaries are stress-controlled and maintain a constant value for the lateral stresses ($\sigma_{1}=\sigma_{2}=100$ kPa). Various criteria have been considered to quantify the RVE size, including having a more homogeneous force path network, having a smother stress–strain diagram, having a repetitive shear behavior, and having a higher chance of capturing the strain localization. Below, they will be explained in detail.

#### 3.1.1. First Criterion: Having a More Homogeneous Force Chain Network

**Figure 7:**
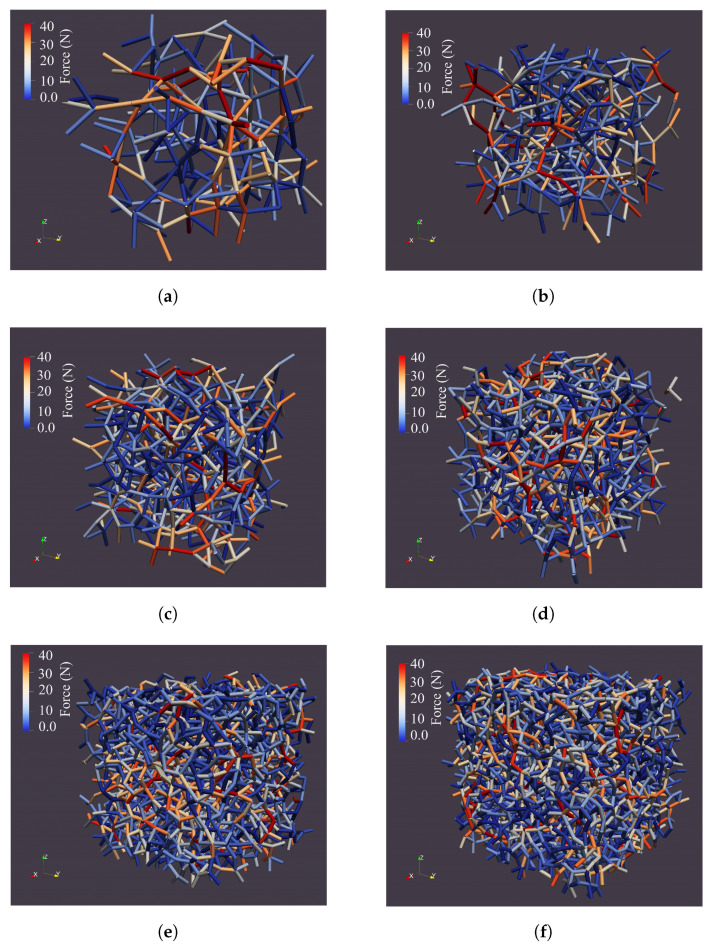
Force chain network for the RVEs with (**a**) 150, (**b**) 300, (**c**) 500, (**d**) 1000, (**e**) 2000, and (**f**) 3000 particles and the periodic boundary conditions.

All of the particles will not participate equally in the deformation of the granular materials. However, when the forces between the particles are more symmetrical, the mechanical behavior of the material will be closer to the bulk state. Figure 7 shows the force chain network of RVEs with a different number of particles in which the RVEs are under confining pressure ($P_{0}$ = 100 kPa) and have periodic boundary conditions. To have an accurate explanation for Figure 7, the average of inter-particle forces and the standard deviation of the inter-particle forces are represented in Table 2. It is observed that the average of the inter-particle forces and their standard deviation are almost the same for all of the RVEs. In addition, the results show that increasing the number of particles does not lead to an increase in the inter-particle forces. This can be due to the fact that the stress on the RVEs is the same. Hence, as the number of particles increases, both the boundary areas and the number of particles that apply force to the boundaries increase in order to keep the stresses felt at the boundaries constant. Therefore, this criterion does not lead us to a specific conclusion for selecting the appropriate number of particles in the RVE.

#### 3.1.2. Second and Third Criteria: Smooth the Stress-Strain Curve and Repetitive Behavior

**Figure 8:**
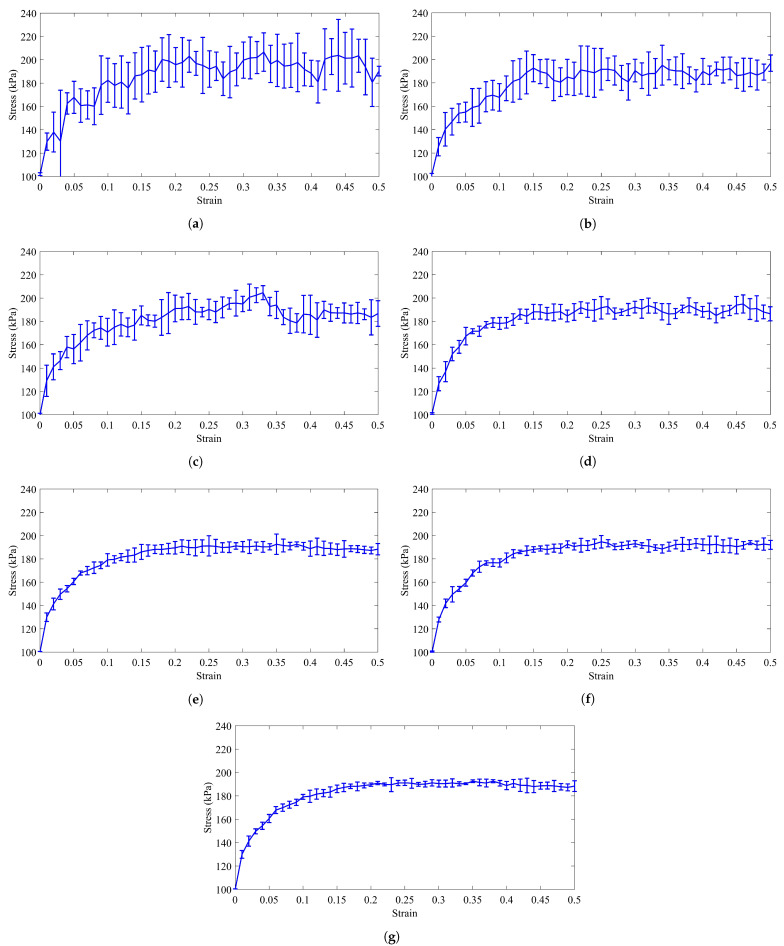
The average of shear behavior of the RVEs with (**a**) 150, (**b**) 300, (**c**) 500, (**d**) 1000, (**e**) 2000, (**f**) 3000, and (**g**) 4000 particles and periodic boundary conditions. The error bars indicate the standard deviation for five times of simulation for each RVE.

For quantifying the smoothness and repetitive behavior, the shear response of the RVEs with a different number of particles is simulated and for each RVE this simulation is repeated five times, and then their average is calculated. Figure 8 shows the average shear behavior of the RVEs with different number of particles, and the error bars represent the standard deviation from the average behavior. The periodic boundary conditions are employed because the bulk behavior of the RVEs is desired. The results show that increasing the number of particles leads to a reduction in the standard deviations and makes the average stress-strain behavior of the RVEs smoother. This is because as the number of particles increases, so does the number of particles taking part in the deformation. Additionally, since the deformation of the granular material is associated with buckling of the force chains and rearrangement of the particles, there are more particles to replace in the new force chains, so that they can withstand the external loads. As a result, fewer stress fluctuations are felt at the boundaries. Therefore, the stress-strain curve will be smoother.

**Figure 9:**
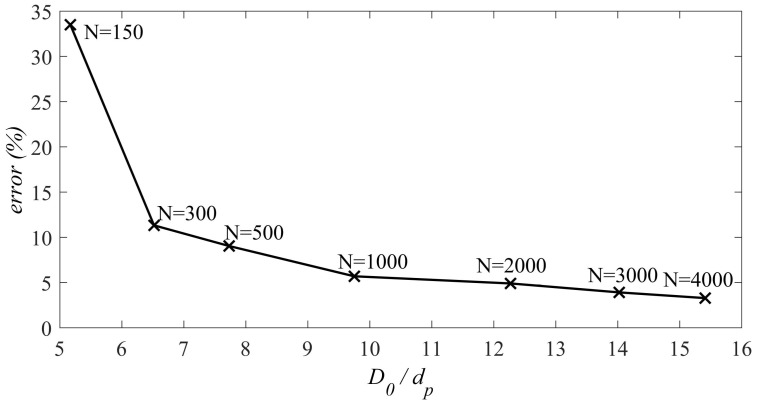
The maximum error of the stress-strain fluctuation versus $D_{0}/d_{p}$ for the different RVEs with the different number of particles.

To quantify this phenomenon, $D_{0}/d_{p}$ is considered in which $D_{0}$ is the size of the RVE at the beginning of the compaction process and $d_{p}$ is the diameter of the particles. Based on [98], for the RVEs with higher $D_{0}/d_{p}$, the fluctuations of the stress-strain diagram are reduced. We define another parameter, in which the ratio of the maximum of the standard deviation to its average stress is considered as the error parameter. The error parameter is as follows:

(18) $$error=\frac{Max(\delta \sigma_{i})}{\sigma_{i}}\times 100\;,$$

where $Max(\delta \sigma_{i})$ is the maximum of the standard deviation and the $\sigma_{i}$ is the average stress that belongs to the maximum standard deviation. In addition, Oda and Kazama [28], by using photoelastic pictures taken from a biaxial test on a two-dimensional assembly of oval rods, indicated that the thickness of localized bands is at least 7 times of the mean particle size. Therefore, the RVEs with 150 and 300 particles in which $D_{0}/d_{p}$ is less than 7 will be refused for this criterion. Moreover, according to Evesque and Adjemian [98], if the number of particles increases, the error will be decreased. In Figure 9, the error parameter is plotted in terms of the parameter $D_{0}/d_{p}$ for the RVEs with different number of particle. For the RVEs with 2000, 3000, and 4000 particles, the error is 4.9%, 3.9%, and 3.28%, respectively. In addition, the parameter $D_{0}/d_{p}$ for the RVEs with 2000, 3000, and 4000 particles is 12.27, 14.023, 15.41, respectively. Therefore, these three RVEs can be considered to be candidates. It is worth mentioning that, to achieve an error of less than 1%, an RVE with at least $10^{7}$ particles must be used [98].

#### 3.1.3. Fourth Criterion: Higher Chance of Capturing the Strain Localization

If the size of the RVE increases, the resolution for capturing the strain localization inside the RVE increases, according to Stroeven et al. [97]. In other words, by increasing the number of particles, the localized zone will be more distinguishable. To examine this issue, the RVEs with the mono-size particles and the solid boundary conditions with the different number of particles are considered. The initial position of particles inside the RVEs is random. The particles are initially compressed by a confining pressure of 100 kPa. While the axial pressure is applied through the upper displacement-controlled boundary ($\dot{\epsilon_{3}}=0.05\;s^{-1}$), the micro-strain is calculated for each particle.

**Figure 10:**
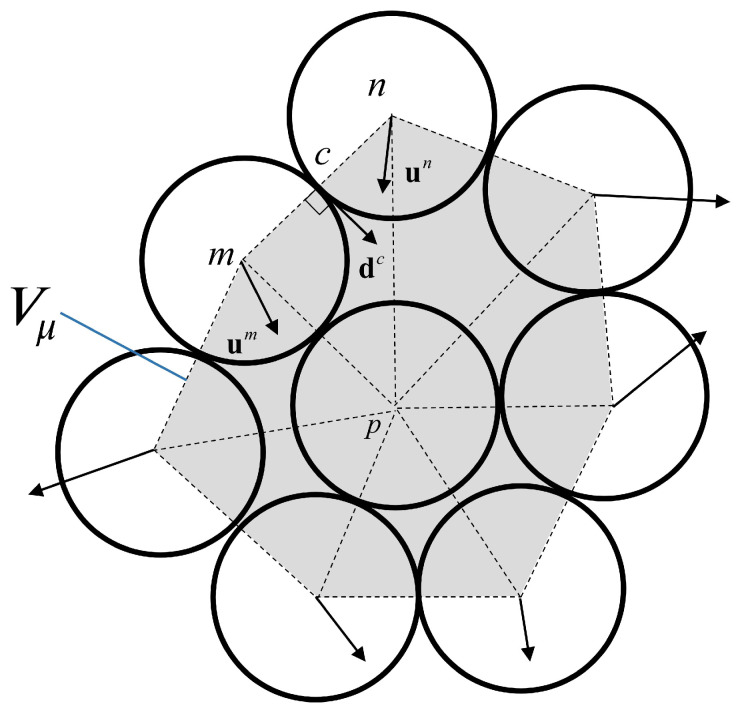
The particle-centered domains for the definition of micro-strain.

The micro-strain tensor for a particle is defined as a function of the displacement of its neighboring (but not necessarily contacting) particles that form a polygonal domain $V_{\mu }$ (Figure 10) [99,100]. This definition is based on a continuous system, which is equivalent to the granular system (see Figure 10). The boundary of this equivalent continuum passes through the center of the surrounding particles. The average displacement gradient in the equivalent continuum, which contains the polyhedral domain $V_{\mu }$, is as follows:

(19) $${\langle \nabla u\rangle }_{\mu }=\frac{1}{V_{\mu }}\underset{V_{\mu }}{\;\int \int \int \;}\frac{\partial (u)}{\partial x}\;dV_{\mu }=\frac{1}{V_{\mu }}\underset{S_{\mu }}{\;\int \int \;}u\cdot n\;dS_{\mu }\;,$$

where $V_{\mu }$ and $S_{\mu }$ are the volume or the surface area of the cell, du is the translation vector of the boundary point, and *n* is the outwards unit normal vector of the boundary of the cell at the same point. In addition, the amount of du for the point *c* is equal to the difference between *m* and *n* nodes translation. Therefore, by applying the $\Delta u^{c}=u^{n}-u^{m}$ and using $d^{c}$, the complementary area vector belonging to the *c* the pair of grains (see [100,101] for more detail), the average displacement gradient for the particle *p* will be:

(20) $${\langle \nabla u\rangle }_{\mu }=\frac{1}{V_{\mu }}\sum_{c}\Delta u^{c}d^{c},$$

and the micro-strain is the symmetric part of Equation (20) and is as follows:

(21) $${\langle \epsilon_{ij}\rangle }_{\mu }=\frac{1}{2V_{\mu }}\sum_{c}\left(\Delta {u_{i}}^{c}{d_{j}}^{c}+\Delta {u_{j}}^{c}{d_{i}}^{c}\right).$$

**Figure 11:**
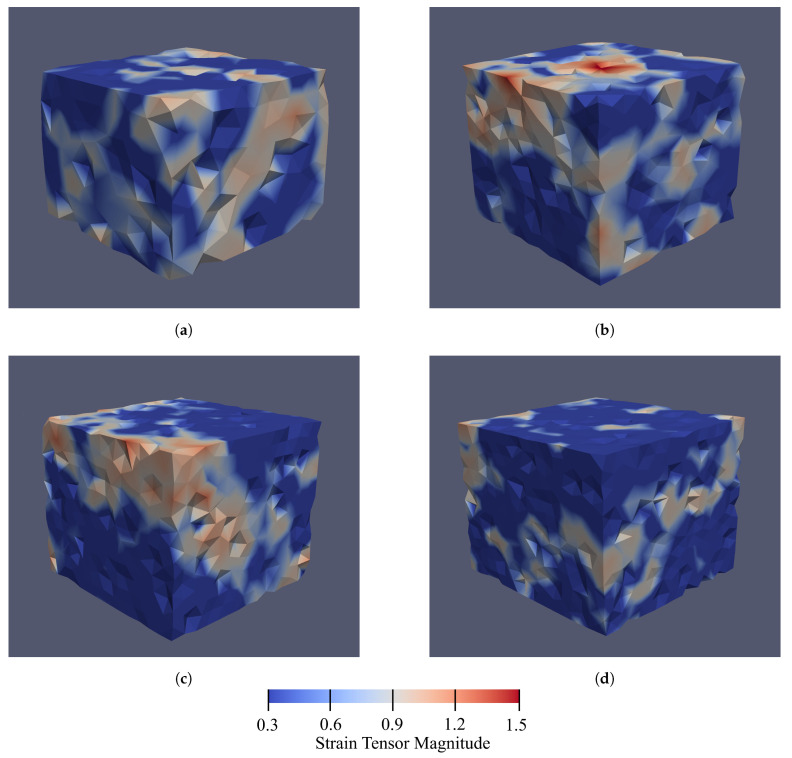
The magnitude of the micro-strain inside the RVEs with (**a**) 1000, (**b**) 2000, (**c**) 3000, and (**d**) 4000 particles.

The micro-strains are visualized for the different RVEs in Figure 11, and it roughly shows that localized areas are more recognizable as the number of particles increases. Hence, as shown in Figure 11, it is easier to detect the localized areas in the RVEs with 3000 and 4000 particles than in the RVEs with 1000 and 2000 particles. However, this judgment is based on the visualization (color difference in Figure 11) and mathematically it could not be cited. Hence, we need a rational criterion to select the RVE with the most probable of the strain localization formation.

As explained in Section 2, the granular material failure is a transition state (a bifurcation) between a quasi-static regime and a dynamic one; consequently, the changing procedure of the kinetic energy could be a reliable indicator of the granular material failure [86,87]. Therefore, by pursuing of the kinetic energy of a granular system, its failure can be recognized. In addition, as Oda and Kazama [28] explained, the particles which are located in the localized zone have the rotation one order of the magnitude more than the rotation of the particles outside of the localized zone. Hence, the onset of failure will be accompanied by a jump in the kinetic energy of the granular system [86]. The kinetic energy of a granular system is:

(22) $$K\left(t\right)=\sum_{i=1}^{N}\left[\frac{1}{2}m^{p}{\left(v^{p}\right)}^{2}+\frac{1}{2}\omega^{p}\left(I^{p}{\omega^{p}}^{T}\right)\right]\;,$$

where $m^{p}$ is the mass of the particle *p*, $v^{p}$ is the linear velocity of the particle *p*, $I^{p}$ is the inertia tensor transformed to the global frame, and $\omega^{p}$ is the angular velocity of the particles *p*. The total number of the particles in each RVE is denoted by N. In view of the fact that the compaction process carried out in the quasi static manner, the kinetic energy of all the RVEs will remain close to zero ($10^{-2}\;\mu J$), except when the failure occurs in them.

**Figure 12:**
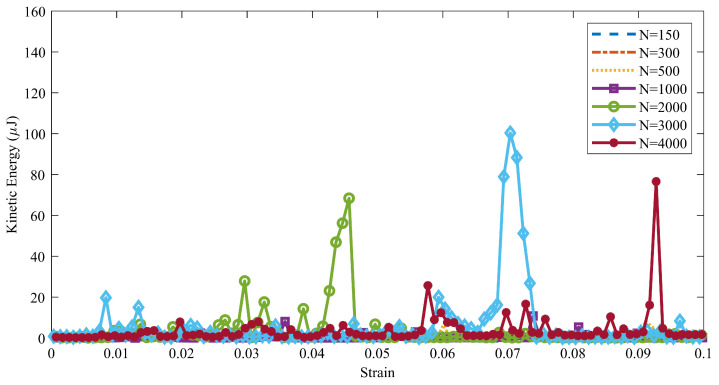
The evolution of the total kinetic energy of different RVEs during the compaction process.

It should be noted that the discrete element method is a dynamic method (in each step, DEM solves Newton’s second law of motion for each particle to find the new interactions and position of particles), hence the initial kinetic energy of the system is not exactly zero (the initial kinetic energy is in the order of $10^{-2}\;\mu J$). Therefore, the outburst of the kinetic energy is an indicator of the higher probability of the failure (localization) in the RVEs. Figure 12 shows the kinetic energy evaluation of the RVEs with a different number of particles. For the RVEs with 2000, 3000, and 4000 particles, the kinetic energy diagram has a jump when the strain equal to 0.044, 0.07, and 0.093, respectively. The local maximums of Figure 12 reveal the bucking of the small force-chains in the RVEs [102]. Therefore, the RVEs with 2000, 3000, and 4000 particles can be treated as candidates.

All of the criteria that are considered in this paper show that, as the number of particles increases, the RVE behavior will be more reliable. On the other hand, increasing the number of particles dramatically affects the computational cost. Therefore, selecting the size of the RVE size is a trade-off between the computational cost and the reliability of the results. The computational cost for DEM simulation is a function of the number of particles, strain rate, the hydrostatic pressure, and the Central Processing Units (CPU) of the system used for the simulation. For example, the computational cost for the RVE with 1000 particles, confining pressure equal to 100 kPa, and the strain rate equal to 0.05 $s^{-1}$ is approximately 10 h. This time for the RVE with 4000 particles is nearly four days. Therefore, the computational cost is the most effective limiting factor for considering more particles. According to our criteria, the error and smoothness of the RVE with 3000 and 4000 particles are almost the same. Hence, the RVE with 3000 particles will be considered for further investigations to reduce the computational cost.

## 4. Failure Analysis

The confining pressure and the speed of compaction process have a significant effect on the final density of the carbon anodes. To investigate the effect of the confining pressure and the strain rate on the failure of the carbon anodes, numerical simulations were conducted on three three-dimensional specimens $S_{1}$, $S_{2}$, and $S_{3}$, which are compacted uniformly by confining pressure equal to 100 kPa, 250 kPa, and 100 kPa, respectively. All of the specimens are cubical in shape and contain 3000 spherical particles of radius 1.87 mm enclosed within six rigid frictionless walls. They were compressed from initially sparse arrangements of the particles to an isotopic state by moving the six rigid frictionless walls until the desired confining pressures are reached. The desired confining pressures for specimens $S_{1}$ and $S_{3}$ are $\sigma_{1}=\sigma_{2}=\sigma_{3}=100$ kPa and for specimen $S_{2}$ is $\sigma_{1}=\sigma_{2}=\sigma_{3}=250$ kPa. They are then subjected to a drained conventional triaxial compression loading path.

The specimens are loaded by applying a constant strain rate in the axial direction, while the stresses are kept constant and equal to confining pressures in the lateral directions. The axial strain rate for specimen $S_{1}$ and $S_{2}$ is ${\dot{\epsilon }}_{3}=0.05\;s^{-1}$, for specimen $S_{3}$ is ${\dot{\epsilon }}_{3}=0.15\;s^{-1}$. The initial porosity of both specimens $S_{1}$ and $S_{3}$ are the same and equal to ϕ=0.466. The initial porosity of the specimen $S_{2}$ is equal to ϕ=0.463. It should be noticed that porosity is defined as $\phi =\frac{V_{T}-V_{s}}{V_{T}}$, in which $V_{s}$ is the volume of spheres and $V_{T}$ is the total volume of specimen.

**Figure 13:**
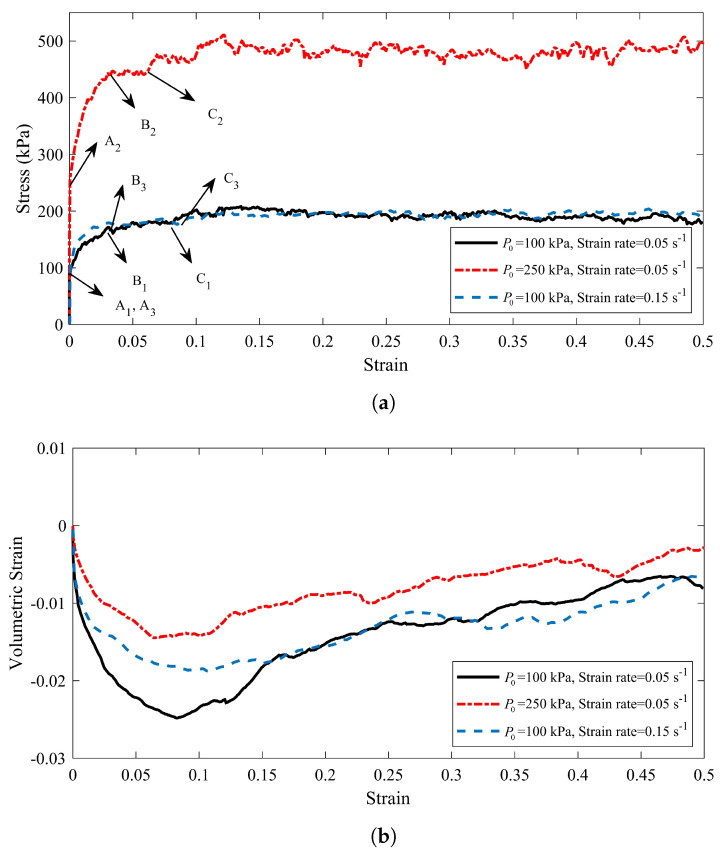
(**a**) Theshear stress behavior and (**b**) the volumetric strain behavior of specimens $S_{1}$, $S_{2}$, and $S_{3}$.

The evolution of both the axial stress $\sigma_{3}$ and the volumetric strain $\epsilon_{v}$ versus the axial strain $\epsilon_{3}$ are shown in Figure 13a,b, respectively, for all three specimens. For specimen $S_{1}$, the axial stress increases continuously (positive hardening regime) toward a limit plateau at which $\sigma_{3}=203$ kPa, and its volumetric strain increases when the strain reaches to 0.0825. By increasing the confining pressure for specimen $S_{2}$, the hardening regime augments and its axial stress increases, until it reaches to the strain $\epsilon_{3}=0.122$. The maximum of the axial stress at this strain is $\sigma_{3}=511$ kPa. Its volumetric strain grows after axial strain reaches to $\epsilon_{3}=0.067$. The shear behavior of specimen $S_{3}$ is similar to specimen $S_{1}$, except that the hardening regime for specimen $S_{3}$ is shorter than specimen $S_{1}$ and it reach to its maximum level of stress when the axial strain is equal to 0.0365. Moreover, the reduction of volumetric strain for specimen $S_{3}$ is less than specimen $S_{1}$, and it attains its minimum value at the axial strain $\epsilon_{3}=0.92$. These analyzes are based on the behavior of the granular material at the boundaries. Although our information in the laboratory experiments is also based on the information which are obtained from the boundaries, when the granular materials fails, the information at the boundaries does not properly delineate the behavior of the material. Therefore, the second-order work analysis requires examining the behavior of the specimens at their critical points.

### 4.1. Second-Order Work from Macroscopic Variables

In Section 2, the two distinct formulations of the second-order work have been reviewed. It was shown by [38] that the semi-Lagrangian and the Eulerian expressions of the second-order work are equivalent as long as the deformation is quasi static. In addition, the second-order work for a granular material can be calculated using by either macroscopic variable or inter-particle variables (microscopic variables) [35]. Ref. [38] shown that the macroscopic second-order work Equation (1) (the variables that are measured at the boundaries) and the microscopic expression (which takes into account the forces between the particles and the micro displacement gradient) are in good agreement. Therefore, in this paper, the Eulerian expression of the second-order work with macroscopic variable will be used.

In order to compute the second order work from the macroscopic variables, three stress states that are defined by their deviatoric stress ratio $\eta =\left(3\left(\sigma_{3}-\sigma_{1}\right)/\left(\sigma_{1}+\sigma_{2}+\sigma_{3}\right)\right)$ are considered (represented by the points ($A_{1},B_{1},C_{1}$), ($A_{2},B_{2},C_{2}$), and ($A_{3},B_{3},C_{3}$) in Figure 13a for specimens $S_{1}$, $S_{2}$, and $S_{3}$, respectively). These arbitrary stress states are chosen before the maximum stress condition (Mohr–Coulomb condition) is reached (see Table 3). In particular, $A_{1}$, $A_{2}$, and $A_{3}$ correspond to the isotropic state for each specimen. The strain states which are specified in Table 3 will constitute initial states on which stress probes (as first introduced by [103]) are performed. It should be noted that, due to frictionless boundaries of specimens and the fact that lateral stresses are kept equal, the stress probe will be written as:

(23) $$\Delta \vec{\sigma }=\left\|\Delta \vec{\sigma }\right\|\left(\cos\left(\alpha \right)\vec{e_{1}}+\cos\left(\alpha \right)\vec{e_{2}}+\sin\left(\alpha \right)\vec{e_{3}}\right)\;.$$

By exposing this stress probe to the specimens, the strain response will be directly obtained from DEM as:

(24) $$\Delta \vec{\epsilon }=\left(\|\Delta \vec{\epsilon_{1}}\|\right)\vec{e_{1}}+\left(\|\Delta \vec{\epsilon_{2}}\|\right)\vec{e_{2}}+\left(\|\Delta \vec{\epsilon_{3}}\|\right)\vec{e_{3}}\;.$$

**Figure 14:**
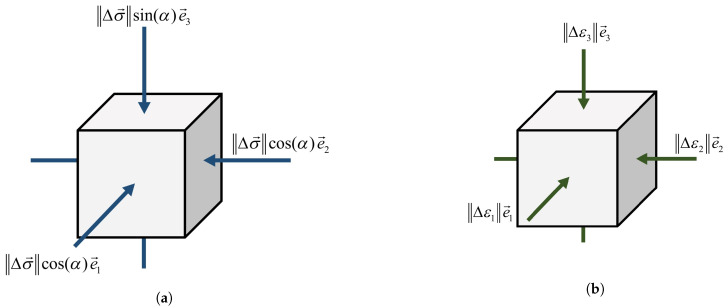
(**a**) The stress probe is applied on the specimens, (**b**) the strain response of the specimens is calculated by DEM.

Because the components of the stress probe are equal in the lateral direction, it can be represented by two values, the norm of the stress probe, $\|\Delta \vec{\sigma }\|$, and an angle, α, which shows the angle between the stress probe vector and the plane perpendicular to the axial direction. Figure 14 shows the components of stress probe applied to the specimen and its strain response. The norm of the stress probe is assumed to be 10 kPa. The angle α is increased from $0^{{}^{\circ}}$ to $360^{{}^{\circ}}$ by increments of $10^{{}^{\circ}}$ to check different stress directions. The maximum axial strain rate for applying the stress probe for specimens $S_{1}$ and $S_{2}$ is equal to 0.05 $s^{-1}$, and for the specimen $S_{3}$ is equal to 0.15 $s^{-1}$. The corresponding strain response vector, $\Delta \vec{\epsilon }$, for each value of the angle α is then calculated by DEM. Subsequently, by using the Eulerian expression of Equation (1), the normalized form of the second-order work associated with each angle α is calculated, as follows:

(25) $$d^{2}\bar{W}=\frac{\Delta \vec{\sigma }\cdot \Delta \vec{\epsilon }}{\|\Delta \vec{\sigma }\|\|\Delta \vec{\epsilon }\|}.$$

**Figure 15:**
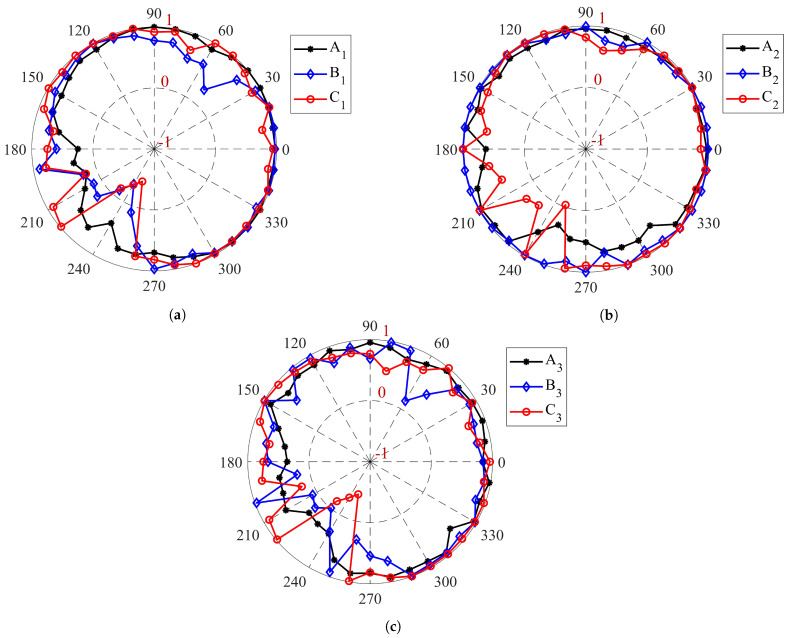
Circular diagrams of the normalized second-order work of (**a**) specimen $S_{1}$ (confining pressure $P_{0}=100$ kPa and strain rate $\dot{\epsilon_{3}}=0.05\;s^{-1}$), (**b**) specimen $S_{2}$ (confining pressure $P_{0}=250$ kPa and strain rate $\dot{\epsilon_{3}}=0.05\;s^{-1}$), and (**c**) specimen $S_{3}$ (confining pressure $P_{0}=100$ kPa and strain rate $\dot{\epsilon_{3}}=0.15\;s^{-1}$) for different values of η.

It is worth mentioning that the value of normalized second-order work is in the range of [−1,1]. Figure 15 represents the value of the normalized second-order work for the specimens $S_{1}$, $S_{2}$, and $S_{3}$ at their critical stress state. The dashed circles shown in Figure 15 demonstrate the zero value for the second-order work. Therefore, when $d^{2}\bar{W}$ is negative the plot is inside the dashed circles, whereas plot is outside the dashed circles for positive values of $d^{2}\bar{W}$.

All of the specimens have a positive second-order work in the isotropic stress state (points $A_{1}$, $A_{2}$, and $A_{3}$). In the other words, all of the specimens are in the stable stress state at the begging of the compaction process. For the specimen $S_{1}$, the cone of the unstable stress directions (inside the dashed circle zone in Figure 15a) are found for $\sigma_{3}=173.5$ kPa when its correspond α is in the range of [225${}^{{}^{\circ}}$, 248${}^{{}^{\circ}}$]. In addition, the stress states of point $C_{1}$, in which the tangent of the volumetric strain diagram (Figure 13b) is zero, are unstable when α in the range of [227${}^{{}^{\circ}}$, 254${}^{{}^{\circ}}$]. By increasing the confining pressure for specimen $S_{2}$ to $P_{0}=250$ kPa, all of the stress states associated with point $B_{2}$ are stable. Moreover, the unstable stress is discovered for the $\sigma_{3}=445$ kPa when its corresponding α is in the range of [249${}^{{}^{\circ}}$, 251${}^{{}^{\circ}}$]. The results indicates that by enhancing the confining pressure, the stable zone for the compaction process increases, and the specimen could be tolerated more stress without any failure inside. In a similar way, by increasing the strain rate to $\dot{\epsilon_{3}}=0.15$ $s^{-1}$, the cone of the unstable stress directions are found when the axial stress is equal to 174.1 kPa (Figure 15c). The unstable corresponding α for this stress state is in the range of [229${}^{{}^{\circ}}$, 231${}^{{}^{\circ}}$]. By comparison the range of the unstable α for the points $B_{1}$ and $B_{3}$ reveals that the unstable zone diminishes when the strain rate enhances. However, by analyzing the response of the stress state at the point $C_{3}$, the unstable stress directions are detected when the range of α is [226${}^{{}^{\circ}}$, 253${}^{{}^{\circ}}$], which is almost similar to the range of the unstable α for the point $C_{1}$ of the specimen $S_{1}$. The second-order work criterion does not specify the instability mode of specimens, as we discussed in Section 2. Therefore, the micro-strain contours are plotted during the compaction process to identify which type of failure modes (localization or diffusing failure) is happened inside the specimens.

### 4.2. Failure Mode along the Drained Compression Path

The evidence of failure in the granular system can be seen when the system exceeds the general stress limit. This evidence for the strain localization failure is in the form of localized bands and unloading areas. While, in the diffuse failure, no specific pattern can be seen [104]. Diagnosis of failure mode in the granular materials, in general, requires special laboratory equipment such as X-ray tomography. While the discrete element method enables us to numerically observe the evolution of the failure state in a specimen. Therefore, thanks to the use of micro-strain contours inside the specimens, the mode of failure inside the specimen can be detected according to the stress-strain state on its boundaries.

**Figure 16:**
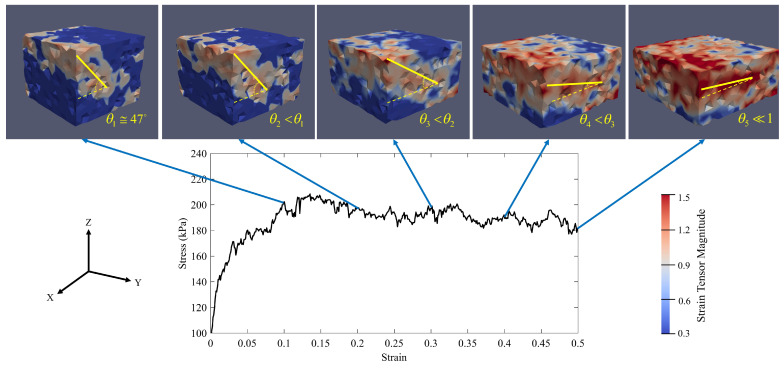
The evolution of micro-strain of specimen $S_{1}$ during the compaction process according to its stress-strain diagram ($P_{0}=100$ kPa and $\dot{\epsilon_{3}}=0.05$$s^{-1}$, $\theta_{i}$ = the angle between the localized band and the maximum principal stress plane).

Figure 16 represents the evolution of the micro-strain of specimen $S_{1}$ during the axial compaction. As discussed in the previous section, specimen $S_{1}$ fails when the axial stress and the axial strain are equal to 173.5 kPa and 0.0413, respectively. At the beginning of the compaction, the micro-strain inside the specimen is uniform. By increasing the compaction in the z-direction, the micro-strain localizes in the specimen. Because the initial angle between the localized band and the the maximum principal stress plane (here XY-plane) is not zero ($\theta_{1}≅47^{{}^{\circ}}$), there are shear stresses within the localized zone. It means that the localized zone is a shear band. By increasing the compaction, the angle decreases to a value that is very close to zero ($\theta_{5}≃0$). The zero angle means that there is no shear stress in the localized band. Hence, the localized band is a compaction band at the end of the compaction. In other words, the shear band becomes the compaction band. These results are consistent with the results of Das et al. [105]. Hence, the compaction parameters (here the confining pressure, $P_{0}$, and the axial stress rate, $\dot{\epsilon_{3}}$) for specimen $S_{1}$ will lead to the formation of a compaction band in the specimen. Therefore, given what has been discussed previously, these compaction parameters will create a compaction band that is prone to horizontal crack formation.

**Figure 17:**
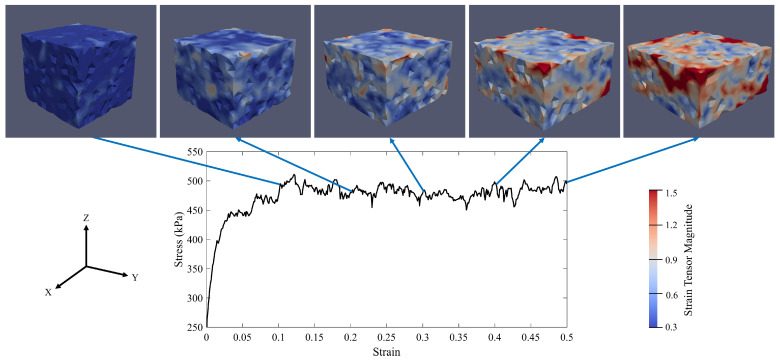
The evolution of micro-strain of specimen $S_{2}$ during the compaction process according to its stress-strain diagram ($P_{0}=250$ kPa and $\dot{\epsilon_{3}}=0.05$$s^{-1}$).

In Figure 17, the micro-strain contours are depicted for specimen $S_{2}$ during the axial compaction process. The micro-strain contour inside specimen $S_{2}$ reveals that, like specimen $S_{1}$, specimen $S_{2}$ initially has a homogeneous deformation. According to Figure 15b, specimen $S_{2}$ fails when it reach to the axial stress 471 kPa and the axial strain 0.067. From this point on, the deformation of specimen $S_{2}$ is no longer homogeneous. However, due to the micro-strain contours inside the specimen, no specific localization pattern could be seen inside the specimen. Hence, the compaction parameters of specimen $S_{2}$ cause a diffusing failure in it. In addition, by comparing its compaction parameters of specimens $S_{1}$ and $S_{2}$, it could be deduced that when confining pressure increases, the failure mode of the specimen intends to be a diffusing failure. In this case, no compaction band is created and thus the possibility of forming a susceptible area to generate the horizontal cracks will be reduced. It is worth know that the dead-weight of the vibro-compactor in the anode production indicates the confining pressure. Consequently, enhancing the dead-weight of the vibro-compactor can be used as a proposed solution to prevent the strain localization in the carbon anodes.

**Figure 18:**
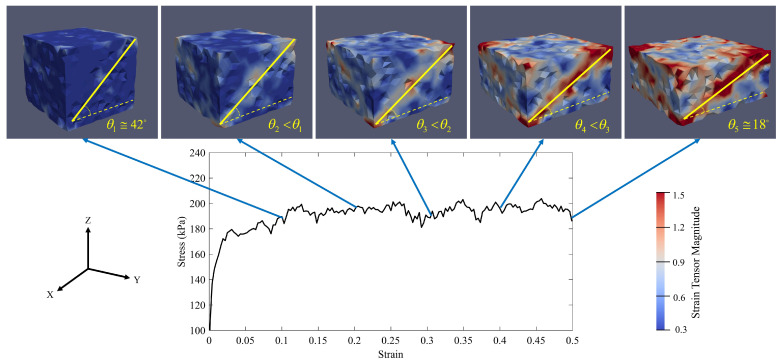
The evolution of micro-strain of specimen $S_{3}$ during the compaction process according to its stress-strain diagram ($P_{0}=100$ kPa and $\dot{\epsilon_{3}}=0.15$$s^{-1}$, $\theta_{i}$ = the angle between the localized band and the maximum principal stress plane).

On the other hand, according to Figure 15c, specimen $S_{3}$ fails when its axial stress and axial strain are equal to 189 kPa and 0.092, respectively. Figure 18 shows that the strain localization mode of failure is predominant in specimen $S_{3}$ and similar to the compaction process of specimen $S_{1}$, the localized band of specimen $S_{3}$ is a type of shear band at the beginning of the compaction process. The angle between the shear band and the maximum principal stress plane (here XY-plane) at the axial strain $\epsilon_{3}=0.1$ is equal to $42^{{}^{\circ}}$. Although the shear band angle ($\theta_{i}$) decreases as the axial strain increases, the shear band remains a shear band at the end of the compaction process ($\theta_{5}≅18^{{}^{\circ}}$). It means that increasing the axial strain rate will postpone the formation of the compaction bands. Therefore, the compaction process can be continued further until the shear band angle reaches close to zero (the shear band turns to a compaction band). Hence, by taking the fact that the vibro-compactor frequency in the anode production process represents the amount of the axial strain rate into account, increasing the frequency can be a suggested solution to inhibit the formation of compaction bands in the anode production process.

## 5. Conclusions

This paper presents a theoretical aspect of the failure analysis in the granular material and a numerical investigation to find the failure in the mono-sized spherical coke aggregate under different compaction conditions. Some conclusions can be summarized, as follows:

- It has been shown that the strain localization could happen in the carbon anodes during the compaction process and if this localized zone is a type of the compaction band, it could be responsible for the crack generation under the stub-holes in the carbon anodes. Because the carbon anode paste behavior during the compaction process is too complex for consideration, the dry mono-sized spherical coke aggregates have been examined.
- When considering failure as a bifurcation from a quasi-static regime to a dynamical one, a failure criterion was inferred, and the notion of the bifurcation domain was specified. The relationship between the kinetic energy of the granular materials and the internal and external second-order work has been evolved. It has been shown that when the failure occurred, the stresses that sense at the boundaries cannot reflect the real stress inside the material.
- Using the DEM simulation, the optimum number of particles which could represent the bulk material for the failure analysis is justified. Four criteria, including having a more uniform force path network, having a smother stress-strain diagram, repetitive behavior of the RVE, and a higher chance of the capturing the strain localization, have been exploited. It has been proved that the RVE with 3000 particles could represent the bulk material behavior in failure analysis.
- The second-order criterion was used for finding the failure threshold in the specimens. The evolution of the shear band to the compaction band was investigated. Moreover, the effect of the confining pressure and the strain rate on the failure of the specimens have been studied. It revealed that, by enhancing the confining pressure, the failure mode of the specimen would be of the diffusing type. However, by increasing the strain rate, the mode of the failure would be the localized type. In addition, the strain rate could postpone the formation of the compaction band, which can generate a susceptible area for the crack generation. The results highlighted that increasing the confining pressure and the axial strain rate could be suggested solutions for preventing the localization or postponing of the formation of the compaction bands in the carbon anode.

This article focuses on the study of the failure behavior of the dry mono-sized coke aggregates. However, the coke aggregates are very complex, as they are composed of particles of different sizes, different shapes, different materials, etc. In the next step, the role of the size distribution and particle shape on the failure of the coke aggregates will be explored by using DEM simulation.

## Figures and Tables

**Table 1:** Coke properties which are used in the discrete element method (DEM) model [5].

Radii (mm)	Density (kg/m${}^{3}$)	Elastic Modulus (MPa)	Poisson Ratio	Friction Angle (rad)	Damping Ratio
1.87	1377	681	0.3	0.31	0.4

**Table 2:** The average of inter-particle forces and their standard deviation for the different size of the representative volume elements (RVEs) RVEs.

Number of the Particles in the RVE	Average Force (N)	Standard Deviation (N)
150	13.72	10.68
300	12.65	9.94
500	12.84	10.55
1000	12.76	9.69
2000	12.71	10.13
3000	12.49	9.39
4000	14.53	9.87

**Table 3:** Deviatoric stress ratio η and axial strain $\epsilon_{3}$ corresponding to the critical points of specimens $S_{1}$, $S_{2}$, and $S_{3}$.

	Specimen $S_{1}$			Specimen $S_{2}$			Specimen $S_{3}$		
	$A_{1}$	$B_{1}$	$C_{1}$	$A_{2}$	$B_{2}$	$C_{2}$	$A_{3}$	$B_{3}$	$C_{3}$
$\epsilon_{3}$	0	0.0413	0.0833	0	0.0365	0.067	0	0.04	0.092
η	0	0.69	0.74	0	0.62	0.76	0	0.65	0.75

## Data Availability

No new data were created or analyzed in this study. Data sharing is not applicable to this article.

## Nomenclature

ϵ	the strain tensor
σ	the Cauchy stress tensor
${\langle \epsilon \rangle }_{\mu }$	micro-strain tensor
$d^{c}$	complementary area vector belonging to the *c* the pair of grains ($m^{2}$)
F	the deformation gradient tensor
P	the first Piola-Kirchhoff stress tensor
u	displacement vector (m)
v	velocity vector (m/s)
X	position vector in material configuration
x	position vector in spacial configuration
K	kinetic energy (J)
$\omega^{p}$	angular velocity of particle (rad/s)
ϕ	porosity
$P_{0}$	confining pressure (Pa)
$\theta_{i}$	the angle between the localized band and the maximum principal stress plane (${}^{{}^{\circ}}$)
φ	friction angle (rad)
$A_{i}$	the area of the surface perpendicular to the direction of $e_{i}$ ($m^{2}$)
*C*	current configuration
$C_{0}$	initial configuration
$d^{2}\bar{W}$	the normalized second-order work
$d^{2}W$	the second-order work (J)
$D_{0}$	initial size of RVE (m)
$d_{p}$	diameter of particle (m)
$f_{i}$	external force in the direction of $e_{i}$ (N)
$\delta_{n}$	overlap at the contact point (m)
$\delta_{t}$	incremental tangential displacement at the contact point (m)
$I^{p}$	inertia tensor transformed to the global frame(kg$m^{2}$)
$K_{n}$	tangential spring stiffness (N/m)
$K_{t}$	normal spring stiffness (N/m)
$m^{p}$	mass of particle (kg)
*S*	surface boundary in current configuration ($m^{2}$)
$S_{0}$	surface boundary in initial configuration ($m^{2}$)
*t*	time (s)
N	number of particle
$T_{i}$	external stress in the direction of $e_{i}$ (Pa)
*V*	volume in current configuration ($m^{3}$)
$v^{p}$	linear velocity of particle (m/s)
$V_{0}$	volume in initial configuration ($m^{3}$)
$V_{\mu }$	volume of polyhedral domain ($m^{3}$)

## Appendix A

Let us consider, at a given time *t*, a homogeneous granular assembly of volume V in equilibrium under prescribed boundary conditions. Then, the rate of the deformation gradient tensor can be obtained as [92]:

(A1) $$\dot{F}=\nabla v\;F.$$

In the other hand, we can use a pull-back transportation to bring the differential from the spatial configuration to the material configuration as:

(A2) $$\nabla v=\frac{\partial v}{\partial X}\;\frac{\partial X}{\partial x}=\frac{\partial v}{\partial X}\;F^{-1}.$$

By substituting Equation (A2) in (A1), it comes:

(A3) $$\dot{F}=\left(\frac{\partial v}{\partial X}\;F^{-1}\right)F=\frac{\partial v}{\partial X}\;.$$

Then, the mean value of the rate of the deformation gradient tensor ($\langle {\dot{F}}_{ij}\rangle$) by using Green formula is equal to:

(A4) $$\langle {\dot{F}}_{ij}\rangle =\frac{1}{V_{0}}\left(\underset{V_{0}}{\;\int \int \int \;}\frac{\partial v_{i}}{\partial X_{j}}dV_{0}\right)=\frac{1}{V_{0}}\left(\underset{S_{0}}{\;\int \int \;}v_{i}N_{i}dS_{0}\right)=\frac{A_{i}{\dot{u}}_{i}}{V_{0}}\;.$$

## Appendix B

By using a similar process, we can calculate the second rate of the deformation gradient tensor by using a time derivative of Equation (A3):

(A5) $$\ddot{F}=\frac{\partial }{\partial t}\left(\frac{\partial v}{\partial X}\right)$$

Because X is independent of *t*, it can be written:

(A6) $$\ddot{F}=\frac{\partial }{\partial X}\left(\frac{\partial v}{\partial t}\right)\;.$$

Then, by using Green formula, the mean value of the second rate of the deformation gradient tensor ($\langle {\ddot{F}}_{ij}\rangle$) is equal to:

(A7) $$\langle {\ddot{F}}_{ij}\rangle =\frac{1}{V_{0}}\left(\underset{V_{0}}{\;\int \int \int \;}\frac{\partial }{\partial X_{j}}\left(\frac{\partial v_{i}}{\partial t}\right)dV_{0}\right)=\frac{1}{V_{0}}\left(\underset{S_{0}}{\;\int \int \;}\left(\frac{\partial v_{i}}{\partial t}\right)\cdot N_{j}\;dS_{0}\right)=\frac{A_{i}{\ddot{u}}_{i}}{V_{0}}\;.$$
